# BMSCs pre-treatment ameliorates inflammation-related tissue destruction in LPS-induced rat DIC model

**DOI:** 10.1038/s41419-018-1060-5

**Published:** 2018-10-03

**Authors:** Biao Wang, Shuming Wu, Zengshan Ma, Tao Wang, Changyong Yang

**Affiliations:** Department of Cardiovascular Surgery, Qilu Hospital of Shandong University, Wenhua West Road, Jinan, Shandong China

## Abstract

This study aimed to investigate the effect of bone marrow-derived mesenchymal stem cells (BMSCs) on disseminated intravascular coagulation (DIC) model rats and to further explore the underlying mechanism. A rat model of lipopolysaccharide (LPS)-induced DIC was successfully established, as indicated by impaired plasma hemostatic parameters and damaged organ functions in rats. Importantly, pre-treatment with rat allogeneic BMSCs before LPS injection significantly alleviated systemic intravascular coagulation, reduced plasma levels of organ dysfunction indicators and pro-inflammatory cytokines, suppressed fibrin microthrombi formation, ameliorated liver, heart, and renal injuries, and increased 24-hour survival rates in LPS-induced DIC rats. The protection of BMSCs against DIC was in a moderately dose-dependent manner. Further investigation revealed that BMSCs co-cultured with peripheral blood mononuclear cells (PBMCs) significantly inhibited the LPS-stimulated PBMCs proliferation and the release of pro-inflammatory cytokines from PBMCs. Of note, upregulation of immunosuppressive factors including indoleamine 2,3-dioxygenase and interleukin-10, which was induced by interferon-γ, contributed to BMSCs-mediated inhibition of LPS-stimulated PBMCs proliferation. These effects do not depend on the direct cell–cell contact. In conclusion, BMSCs pre-treatment ameliorates inflammation-related tissue destruction in LPS-induced DIC model rats. The protection of BMSCs may be attributed to their anti-inflammatory and immunomodulatory properties, which render BMSCs a promising source for stem cell-based therapeutic approaches in inflammation-related DIC.

## Introduction

Disseminated intravascular coagulation (DIC) is a devastating clinical condition caused by unbalanced activation between coagulation and fibrinolysis^1^. It typically occurs as an acute complication in patients with underlying life-threatening illnesses, such as severe infection, severe sepsis, solid or hematologic malignancies, severe trauma, placental abruption, and obstetric calamities^2^. DIC is characterized by systemic activation of coagulation, potentially leading to thrombotic obstruction of small and midsize vessels, thereby contributing to multiple organ failure (MOF)^3^. MOF involves disturbed microcirculation owing to the formation of numerous microthrombi in several organs and is one of the most common complications of DIC^4^. The pathophysiology of DIC is multifactorial involving intertwined feedback loops between the coagulant, immune, and inflammatory pathways^5^. Although DIC is a serious disease, there is no gold standard for its diagnosis, no single biomarker by which DIC can be clearly diagnosed, and no anticoagulants have been recommended for the treatment of DIC in worldwide^6^.

Mesenchymal stem cells (MSCs) are multipotent non-hematopoietic progenitor cells that can differentiate into bone marrow stromal cells, adipocytes, osteoblasts, chondrocytes, tenocytes, neurons, skeletal myocytes, and cells of visceral mesoderm^7,8^. MSCs have become promising candidates for the development of novel allogeneic cell-based therapeutic strategies in harnessing inflammation in the repair or regeneration of various damaged tissues^9,10^. Until now, most cell-based therapies were conducted using the well-characterized bone marrow-derived MSCs (BMSCs). Growing evidence has revealed that BMSCs display profound immunomodulatory and anti-inflammatory capacities^11–13^. Furthermore, BMSCs can exert immunosuppressive and anti-inflammatory effects both in vitro and in vivo by inhibiting the proliferation and function of innate and adaptive immune cells, such as natural killer (NK) cells, dendritic cells, and T and B lymphocytes^14–17^. To date, various studies have demonstrated that soluble factors including transforming growth factor β1 (TGF-β1), interleukin-10 (IL-10), cyclooxygenase-2 (COX-2), inducible nitric oxide synthase (iNOS), indoleamine 2,3-dioxygenase (IDO), and hepatocyte growth factor (HGF), either produced constitutively by MSCs or as a result of cross-talk with target immune cells, have been attributed to the immunomodulatory properties of MSCs^18^. Interestingly, the pro-inflammatory cytokine interferon-γ (IFN-γ), secreted by activated T cells, is capable of regulating the immunomodulatory functions of BMSCs via regulation of a variety of immunosuppressive factors, including COX-2, iNOS, IDO, and IL-10^7,19,20^. In addition, the unique immunomodulatory and anti-inflammatory effects of MSCs have been demonstrated in several animal disease models related to inflammation, such as bowel disease^18^, periodontitis^21^, and sepsis^22^. Taken together, these observations have provided convincing evidence that BMSCs-based therapy may be potential for the treatment of DIC.

The aim of present study was to investigate the in vivo effect of BMSCs pre-treatment on inflammation-related DIC rat model induced by lipopolysaccharide (LPS) and to explore whether their anti-inflammatory protection of BMSCs against DIC was associated with their immunomodulatory effect on peripheral blood mononuclear cells (PBMCs) proliferation.

## Materials and methods

### Animals

Adult pathogen-free male Wistar rats (6–7 weeks old, weighing 160–170 g) were obtained from the Laboratory Animal Center of Shandong University (Jinan, Shandong, China). The experimental procedures were approved by the Animal Care and Use Committee of Shandong University. All rats were kept per cage with free access to food and water, and a 12/12 h light/dark cycle, with an ambient temperature of 20–25 °C.

### Isolation and culture of BMSCs

Isolation of BMSCs from Wistar rats was performed according to our previous studies^23^. The morphological characteristics of BMSCs were examined using an inverted microscope. 3-(4,5-Dimethylthiazol-2-yl)-2,5-diphenyltetrazolium bromide assay was performed to draw the growth curve of the BMSCs cultured for 1–8 days as previously described^24^. Flow cytometry and differentiation assays were carried out according to our previous studies^23^ to verify BMSCs based on established criteria^25^. The fourth passage BMSCs were used in subsequent experiments.

### Experimental DIC models

The establishment DIC rat model was performed according to the methods described by our previous studies^23,26^. Rats were anesthetized with an intraperitoneal injection of pentobarbital sodium salt (30 mg/kg, Sigma-Aldrich). Experimental DIC models were treated with sustained intravenous infusion of 3 mg/kg LPS diluted in 1 ml saline for 1 h via the tail vein. The control group was injected with the same volume of saline. The laboratory diagnosis of DIC in rats was performed according to a previous study^27^.

### Pre-treatment with allogeneic BMSCs in rats

Sixty male rats were randomly divided into five treatment groups and one control group (*n* = 10/group). (1) Control: rats were pre-treated with 1 ml culture medium (vehicle of allogeneic BMSCs) and then administered a sustained intravenous infusion of 1 ml normal saline (vehicle of LPS) for 1 h via tail vein; (2) LPS: rats were pre-treated with 1 ml culture medium and then treated with a sustained intravenous infusion of 3 mg/kg LPS diluted in 1 ml saline for 1 h via tail vein; (3) LPS + BMSCs: rats were pre-treated with 1 × 10^3^, 1 × 10^4^, 1 × 10^5^, and 1 × 10^6^ allogeneic BMSCs in 1 ml cultured medium via tail vein for 3 consecutive days (three times at intervals of 24 h) before LPS injection. LPS was injected via tail vein at the end of 24 h after last BMSCs injection.

### Parameter measurement

The blood sample was withdrawn from the abdominal aorta at 0 h (before), 2 h, 4 h, and 6 h after LPS injection. Platelet (PLT) counts were performed with an automated device for animals (Celltac, MEK-5128, Nihon Kohden Co., Tokyo, Japan). d-dimer levels were determined by the quantitative latex agglutination test (Diatron, Tokyo, Japan). Fibrinogen (Fib) levels, activated partial thromboplastin time (APTT), and prothrombin time (PT) were measured using an ACL-9000 Coagulation Analyzer. Plasma levels of creatinine (Cr), alanine aminotransferase (ALT), and creatinine kinase-MB (CK-MB) were measured with commercially available kits (Sigma) with a fully automated clinical chemistry analyzer (Hitachi 912, Boehringer Mannheim, Germany) according to manufacturer’s instructions. Plasma levels of endothelin (ET) were determined with a commercial enzyme-linked immunosorbent assay (ELISA) kit (Wako, Osaka, Japan).

### Histopathology

After experiments, the rats were killed and their organs (including the heart, liver, and kidney) were harvested and prepared for histological studies. A part of the sections were stained with hematoxylin and eosin (HE), and the others were stained with phosphotungstic acid hematoxylin (PTAH) according to the routine staining procedure. Fibrin was red-stained in HE staining and violet blue-stained in PTAH staining. They were examined under a light microscope (Olympus, Japan).

Quantitative evaluation of the heart injury was performed according to a pathologic score system (neutrophil infiltration, hemorrhage, and necrosis) ranging from 0 (normal) to 6 (severe) as previously described^28^. Liver injury of each section was assessed according to Suzuki’s criteria^29^, which accounts for the following three separate criteria: vascular congestion, hepatocyte vacuolization, and necrosis. The scores from 0 (none) to 4 (severe) for each of the three criteria were then combined, leaving a combined liver injury score ranging from 0 (normal) to 12 (severe). Quantitative evaluation of the renal injury was performed based on histological parameters: tubular necrosis, interstitial edema, loss of brush border, and casts formation. The scoring system used was 0, absent; 1, present; and 2, marked, as described previously^30^. A minimum of 10 fields for each section were examined and assigned for the severity of changes. Blinded analysis of the histological samples was performed by two experts (Department of Cardiovascular Surgery, Qilu Hospital of Shandong University).

### Cytokine analysis by ELISA

The levels of IL-1β, IL-10, tumor necrosis factor-α (TNF-α), and IFN-γ in the cell supernatant after 72 h of incubation and plasma levels of TNF-α, IFN-γ, and IL-1β were determined by ELISA kits purchased from R&D Systems (Minneapolis, MN, USA). All conditions were performed in triplicate.

### Isolation of PBMCs

Fresh blood (4 ml) was drawn from the rat inferior vena cava at the end of 24 h after last BMSCs injection. Next, blood was heparinized for the isolation of PBMCs by a Ficoll-Hypaque density gradient centrifugation. In brief, the mixture of lymphocyte isolation liquid (Ficoll, GE Healthcare Bioscience, Piscataway, New Jersey, USA), serum-free RPMI 1640 (Gibco, USA), and fresh blood (1: 1: 1, v/v/v) was centrifuged at 2000 × *g* for 20 min at 25 °C. After that, the mononuclear cell layer (white layer) was carefully aspirated by a syringe and re-suspended in 3 ml of RPMI 1640 medium containing 10% fetal calf serum (FCS, Hyclone, USA). The active PBMCs accounted for 95% after trypan blue dyeing. PBMCs were finally adjusted the concentration of 2 × 10^6^/ml.

### Treatment with allogeneic BMSCs and LPS in PBMCs

PBMCs isolated from Wistar rats were randomly divided into six treatment groups and one control group for co-culture with BMSCs under LPS stimulation.

(1) PBMCs (control): PBMCs (100 μl, 2 × 10^6^/ml) + RPMI 1640 medium/10% FCS (100 μl);

(2) PBMCs + LPS: PBMCs (100 μl, 2 × 10^6^/ml) + RPMI 1640 medium/10% FCS (100 μl) + LPS (final concentration, 0.5 μg/ml, Difco Laboratory, USA);

(3–6) PBMCs + LPS + BMSCs (1 × l0^2^/1 × l0^3^/1 × l0^4^/1 × l0^5^): PBMCs (100 μl, 2 × 10^6^/ml) + BMSCs (corresponding 1 × l0^3^/1 × l0^4^/1 × l0^5^/1 × l0^6^/ml, 100 μl) + LPS (final concentration, 0.5 μg/ml).

### PBMCs proliferation assay

^3^H-TdR incorporation assay was performed to evaluate the proliferation capacity of PBMCs isolated from Wistar rats in co-culture with or without LPS or BMSCs. In brief, PBMCs were plated into 96-well plates (2 × 10^6^/ml, 200 μl/well) and treated with or without BMSCs isolated from rats, IL-10-neutralizing antibody (cat. no MAB417, 10 μg/ml, R&D Systems), TGF-β1-neutralizing antibody (cat. no. 1D11, 10 μg/ml, R&D Systems), indomethacin (a COX-2 inhibitor, cat. no. 9758, 20 μM, Sigma-Aldrich), l-NAME (*N*-nitro-l-arginine methyl ester, an iNOS inhibitor, cat. no. M18168, 1 mM, Meryer, China), 1-MT (1-methyl-l-tryptophan, an IDO inhibitor, cat. no. 860646, 500 μM, Sigma-Aldrich), and antibody IFN-γ (cat. no. ab133566, Abcam, USA) followed by the stimulation of LPS (0.5 μg/ml, Difco Laboratory, USA) or not. After 72 h of incubation, 20 μl ^3^H-TdR (10 μci/ml) was added into each well for 8 h of incubation. PBMCs were harvested onto glass fiber filter paper, and the counts per minute were determined using a Wallac TriLux 1450 MicroBeta microplate scintillation counter (PerkinElmer, Waltham, MA, USA). All conditions were performed in triplicate.

### Western blot

Protein was isolated from BMSCs and subjected to western blot analysis as previously described^18^. Results were analyzed using Alpha View Analysis Tools (AlphaViewSA software version 3.2.2, ProteinSimple, Santa Clara, CA). β-actin served as the loading control.

### Detection of IDO enzymatic activity

IDO activity was analyzed by measuring the concentration of kynurenine in BMSCs culture in response to IFN-γ (PeproTech) or co-culture with PBMCs in the absence or presence of 0.5 μg/ml LPS using a high-pressure liquid chromatography technique according to a previous study^31^.

### Statistical analysis

Data were expressed as mean ± standard deviation from three independent experiments. Statistical analyses were performed using SPSS statistical software package standard version 16.0 (SPSS, Inc., Chicago, IL, USA). Experiments were performed in triplicate. Statistical differences between two independent groups were determined using Student’s *t* test. For multiple groups, the statistical analyses were performed by one-way ANOVA followed by Tukey’s test. Survival analysis was performed by using the Kaplan–Meier method. *P* < 0.05 was considered to indicate a statistically significant difference.

## Results

### Culture and identification of BMSCs from wistar rats

Cultured BMSCs from Wistar rats were observed to be round or oval following inoculation. After a serial passage of adherent cells, a homogeneous population of spindle-shaped cells proliferated to form colonies (Supplementary Figure 1A). BMSCs displayed vigorous growth (Supplementary Figure 1B). The surface makers of the fourth passage BMSCs were analyzed by flow cytometry. The immunophenotypes of these cells were persistently positive for CD29, CD44, CD10, and CD106, but negative for CD34 and CD45 (Supplementary Figure 1C). When exposed to the endothelial medium, the cells expressed the specific marker of von Willebrand factor (Supplementary Figure 1D). Furthermore, the BMSCs differentiated into osteoblastic cells, as judged by their ability to mineralize the extracellular matrix (Supplementary Figure 1E). Moreover, when exposed to an adipogenic stimulus, the cells displayed an adipocyte phenotype and accumulation of neutral lipid droplets in the cytoplasm (Supplementary Figure 1F). These results indicated that the obtained BMSCs conformed to the well-established criteria.

### BMSCs pre-treatment alleviates systemic intravascular coagulation, attenuates organ dysfunction, and reduces pro-inflammatory cytokines

**Fig. 1:**
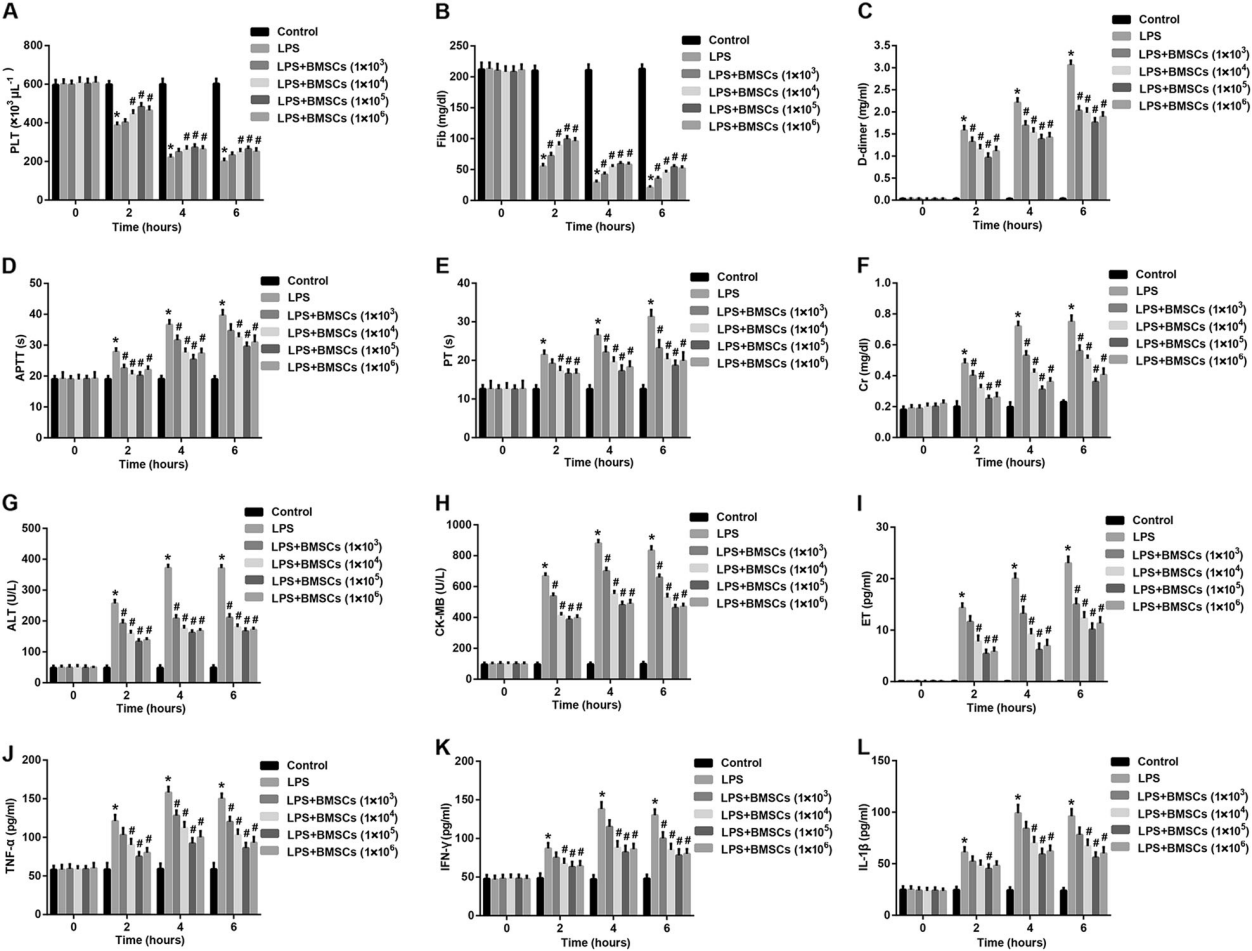
Effect of BMSCs pre-treatment on plasma markers, organ function-associated indicators, and pro-inflammatory cytokines in LPS-induced DIC model rats. Plasma levels of **a** platelet (PLT) counts, **b** fibrinogen (Fib), **c** d-dimer, **d** activated partial thromboplastin time (APTT), **e** prothrombin time (PT), **f** creatinine (Cr), **g** alanine aminotransferase (ALT), **h** creatinine kinase-MB (CK-MB), **i** endothelin (ET), **j** TNF-α, **k** IFN-γ, and **l** IL-1β from the abdominal aorta at 0 h (before), 2 h, 4 h, and 6 h after LPS injection were examined. *N* = 10/group. ^*^P < 0.05, vs. control; ^#^*P* < 0.05, *vs*. LPS

Plasma PLT counts and Fib concentrations in the rats were significantly decreased at 2 h, 4 h, and 6 h after LPS administration as compared to the control group (Fig. 1a, b). In contrast, a simultaneous significant increase of d-dimer, PT, and APTT after LPS administration for 2 h, 4 h, and 6 h was observed (Fig. 1c–e). These changes in plasma markers for DIC demonstrated that the DIC rat model was successfully established. To investigate the effect of BMSCs pre-treatment on systemic intravascular coagulation, we then detected the changes of plasma markers for DIC. Data showed that pre-treatment with BMSCs dramatically increased PLT counts and Fib concentrations as compared with the LPS group (Fig. 1a, b). However, plasma d-dimer levels in the BMSCs pre-treatment groups were significantly decreased compared with those in the LPS group (Fig. 1c). In addition, APTT and PT in LPS-induced DIC rat model group were notably shortened by pre-treatment with BMSCs (Fig. 1d, e). These results indicated that pre-treatment with BMSCs alleviated systemic intravascular coagulation. Meanwhile, this protective effect was moderately BMSCs dose dependent.

To investigate whether BMSCs pre-treatment has the effect to protect the organ function in LPS-induced DIC model, we measured the plasma levels of several organ function indicators. Data revealed that the plasma concentrations of Cr (a marker of renal injury; Fig. 1f), ALT (a marker of liver injury; Fig. 1g), CK-MB (a marker of myocardial injury; Fig. 1h) and ET (a marker of endothelial damage; Fig. 1i) in the LPS group were dramatically increased as compared with the control group, indicating organ damage in LPS-induced DIC model. Importantly, plasma levels of these organ function indicators were greatly reduced in the BMSCs pre-treatment groups compared with the LPS group. The regulatory effect also relied on the infused dose of BMSCs.

To explore the effect of BMSCs pre-treatment on inflammation on DIC, we measured the plasma levels of pro-inflammatory cytokines. We found that plasma levels of TNF-α (Fig. 1j), IFN-γ (Fig. 1k), and IL-1β (Fig. 1l) in the LPS group were dramatically increased as compared with the control group, indicating inflammation activation in LPS-induced DIC model. Importantly, plasma levels of these pro-inflammatory cytokines were greatly reduced in the BMSCs pre-treatment groups compared with the LPS group. This regulatory effect of BMSCs was also presented as dose dependent.

### BMSCs pre-treatment ameliorates tissue destruction in LPS-induced DIC model

Given that DIC may result in MOF, we assessed histological injury in the heart, liver, and kidney from LPS-induced DIC rats by HE and PTAH staining.

### Heart

**Fig. 2:**
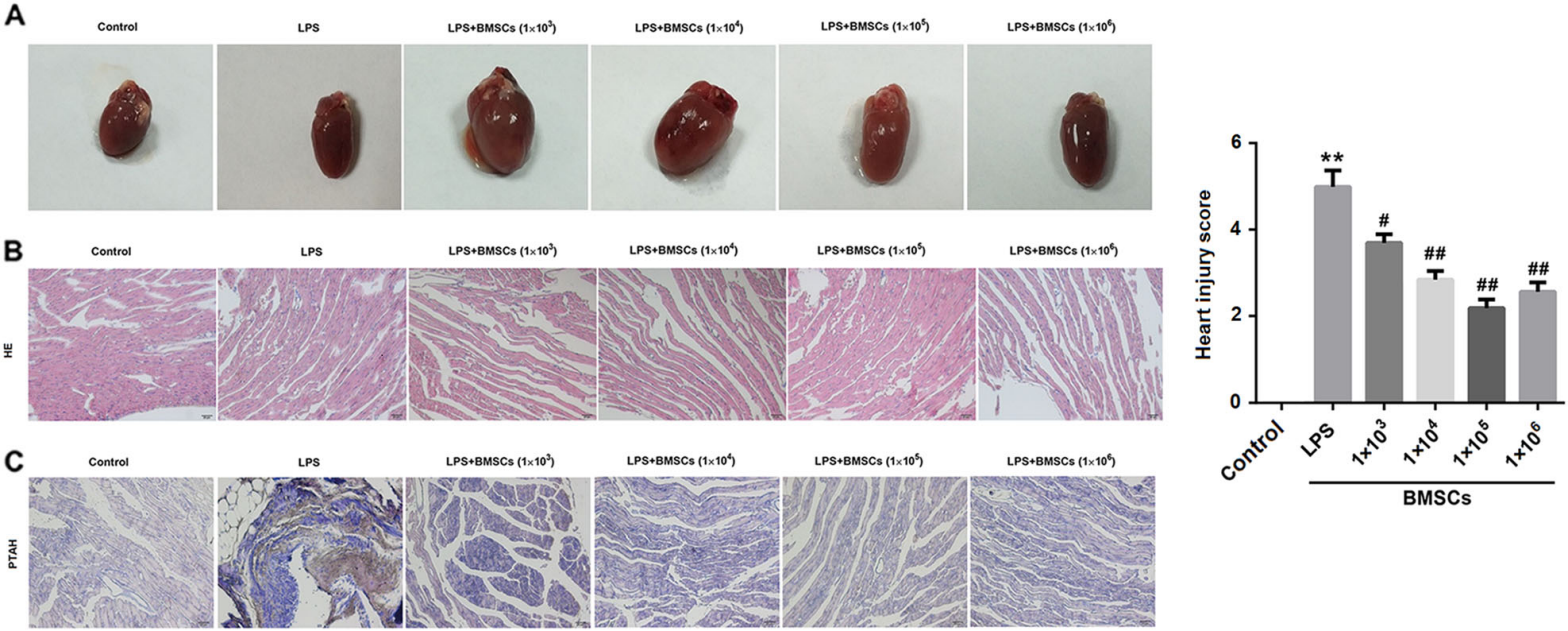
Effect of BMSCs pre-treatment on hearts in LPS-induced DIC model rats. Six hours after LPS injection, the rats were killed and their heart tissue sections were examined by hematoxylin and eosin (HE) and Mallory’s phosphotungstic acid hematoxylin (PTAH) staining. **a** Representative images of heart tissues. **b** Representative images of HE staining of heart tissues from rats. **c** Representative images of PTAH staining of heart tissues from rats. Fibrin was red-stained in HE staining and violet blue-stained in PTAH staining. The bar graphs show the heart injury score. *N* = 10/group.^*^P < 0.05, vs. control; ^#^*P* < 0.05, ^##^*P* < 0.01, vs. LPS

The control heart tissue exhibited the normal appearance of myocardium and clearly identifiable small blood vessels. In contrast, the myocardium was dark red in color and showed severe myocardial edema in the LPS group. In the BMSCs pre-treatment groups, the color and appearance of myocardium were close to that of control heart (Fig. 2a). In addition, as shown in HE and PTAH staining, pre-treatment with BMSCs reduced capillary thrombosis or fibrin clots in the LPS-induced DIC models (Fig. 2b, c). Histological analysis demonstrated pre-treatment with BMSCs led to a significant reduction of heart injury in the LPS-treated animals (Fig. 2).

### Liver

**Fig. 3:**
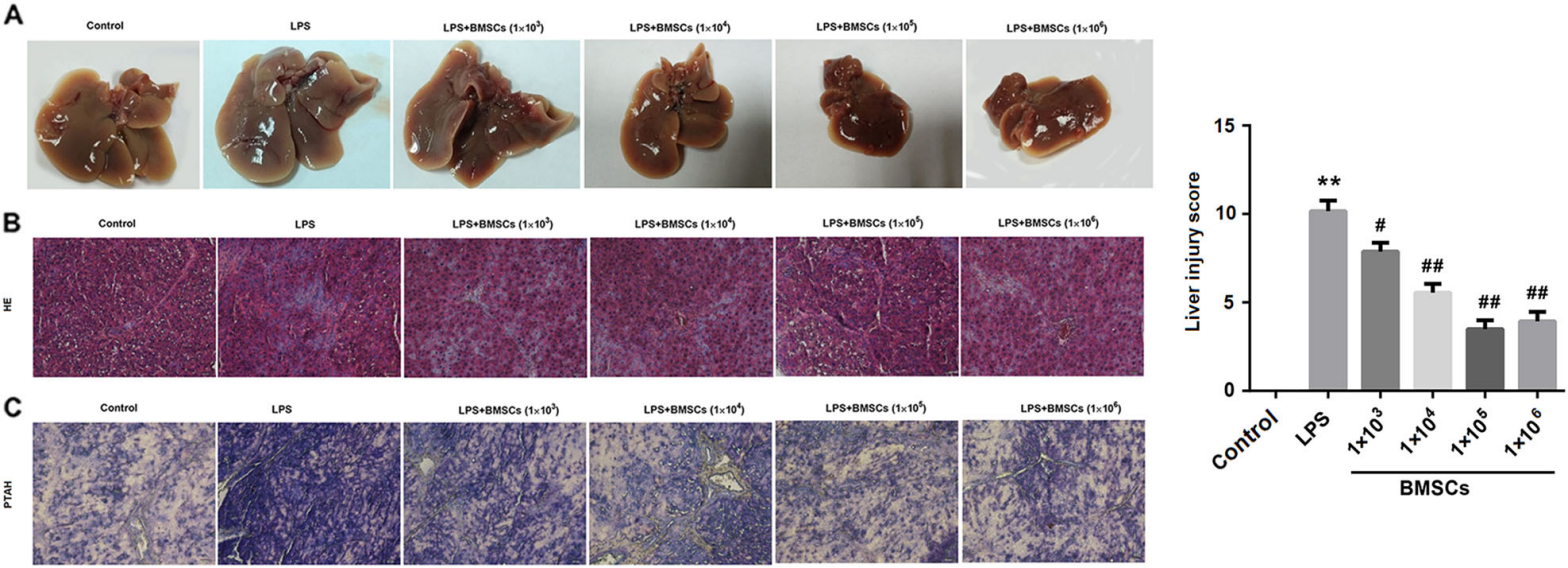
Effect of BMSCs pre-treatment on livers in LPS-induced DIC model rats. Six hours after LPS injection, the rats were killed and their liver tissue sections were examined by hematoxylin and eosin (HE) and Mallory’s phosphotungstic acid hematoxylin (PTAH) staining. **a** Representative images of liver tissues. **b** Representative images of HE staining of liver tissues from rats. **c** Representative images of PTAH staining of liver tissues from rats. Fibrin was red-stained in HE staining and violet blue-stained in PTAH staining. The bar graphs show the liver injury score. *N* = 10/group. ^*^P < 0.05, vs. control; ^#^*P* < 0.05, ^##^*P* < 0.01, vs. LPS

The liver in the control group was normal red in color. As expected, the liver treated with LPS was dark red in color accompanied with some hemorrhagic spots. However, the hepatic color was close to that of control liver after BMSCs pre-treatment (Fig. 3a). Subsequently, HE staining showed the blood was stagnant in hepatic central veins and the hepatocytes were swollen after LPS treatment (Fig. 3b). Furthermore, extensive fibrin microthrombi formation appeared in hepatic lobules shown in PTAH staining (Fig. 3c). However, the injuries were ameliorated by BMSCs pre-treatment (Fig. 3).

### Kidney

**Fig. 4:**
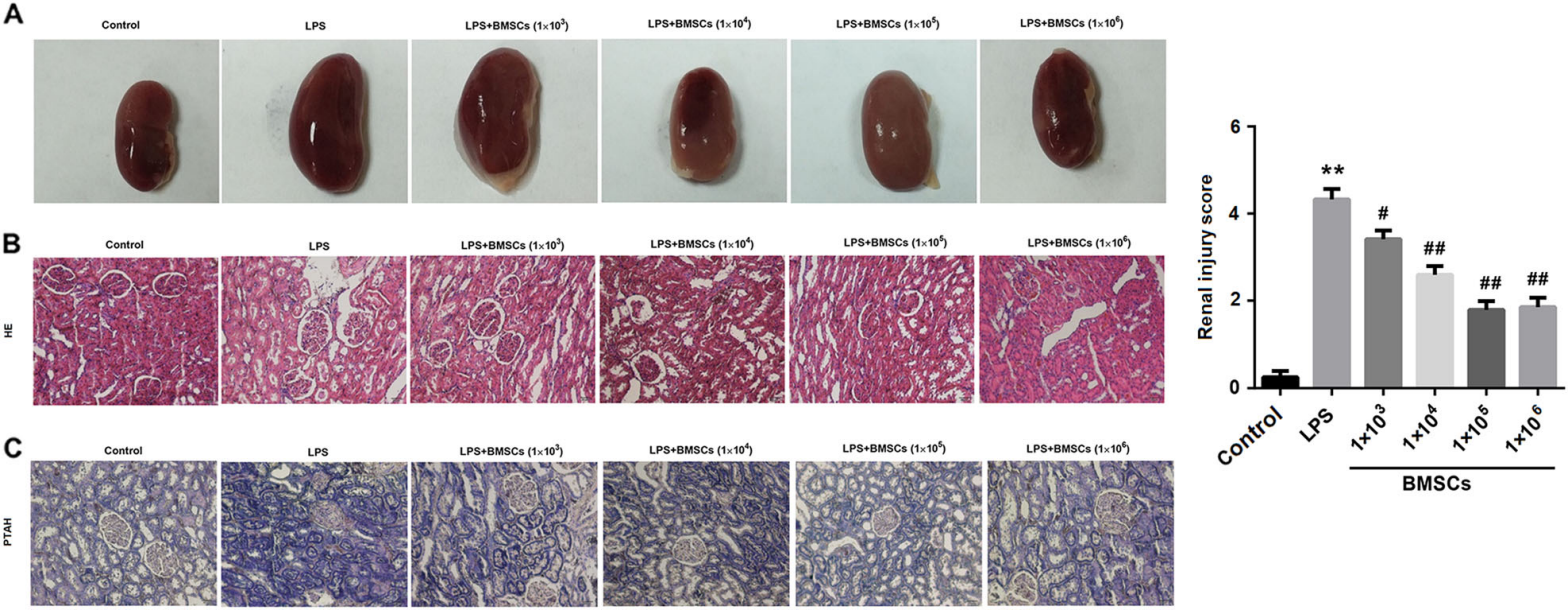
Effect of BMSCs pre-treatment on kidneys in LPS-induced DIC model rats. Six hours after LPS injection, the rats were killed and their renal tissue sections were examined by hematoxylin and eosin (HE) and Mallory’s phosphotungstic acid hematoxylin (PTAH) staining. **a** Representative images of renal tissues. **b** Representative images of HE staining of renal tissues from rats. **c** Representative images of PTAH staining of renal tissues from rats. Fibrin was red-stained in HE staining and violet blue-stained in PTAH staining. The bar graphs show the renal injury score. *N* = 10/group.^*^P < 0.05, vs. control; ^#^*P* < 0.05, ^##^*P* < 0.01, vs. LPS

Inspection of the kidney revealed that the renal color was ruddy in the control group. However, the renal color turned to be dark after LPS treatment. As expected, the renal color was lighter in the BMSCs pre-treatment groups than that in the LPS group (Fig. 4a). HE and PTAH staining of kidneys revealed that BMSCs pre-treatment alleviated the lesions and capillary thrombosis in the LPS-induced DIC rat model (Fig. 4b, c). Histological analysis revealed that pre-treatment with BMSCs significantly reduced the LPS-induced renal injury (Fig. 4).

Our data confirmed the successful establishment of DIC induced by LPS, as evidenced by positive PTAH staining for intravascular fibrin formation. Moreover, BMSCs pre-treatment ameliorated organ dysfunction in LPS-induced DIC rats. In addition, the protective effect of BMSCs on the heart, liver, and kidney was moderately dose dependent.

### BMSCs pre-treatment increases survival rates in LPS-induced DIC model

**Fig. 5:**
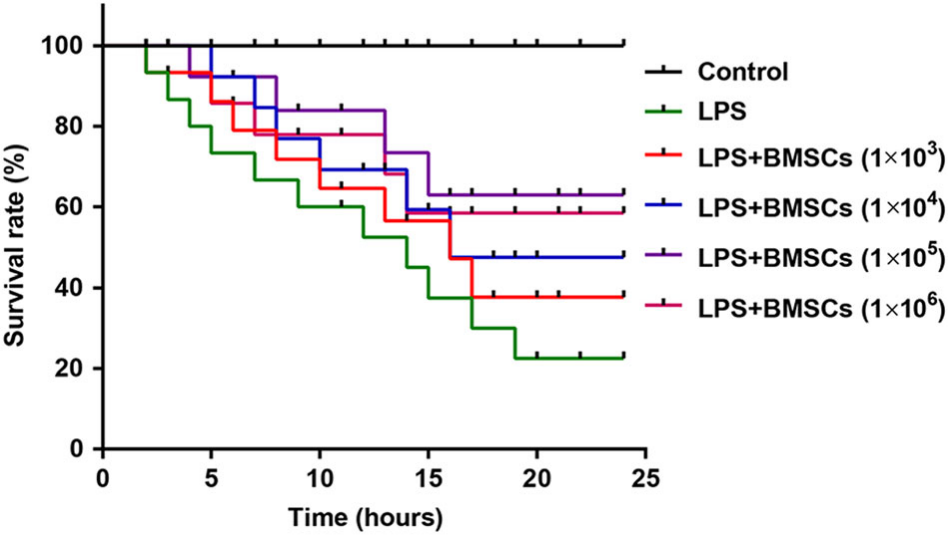
BMSCs pre-treatment increased survival rates in LPS-induced DIC model. Kaplan–Meier survival analysis of the 24-h survival rates among the six groups. The procedures of pre-treatment with allogeneic BMSCs in rats were the same as mentioned above, except that the concentration of LPS was increased from 3 mg/kg to 10 mg/kg. *N* = 10/group

To evaluate the protective effects of BMSCs pre-treatment on LPS-induced DIC rats, we selected another 60 adult male Wistar rats and randomly divided them into six groups. The procedures of pre-treatment with allogeneic BMSCs in rats were the same as mentioned above, except that the concentration of LPS was increased from 3 mg/kg to 10 mg/kg. We observed the survival times of rats in the six groups within 24 h after LPS injection and analyzed these survival data. Data for Kaplan–Meier survival analysis revealed that the 24-h survival rates varied with the different treatments among the six groups. The survival rate in the control group was 100%, and a marked decline of survival rate was observed in the LPS group. However, pre-treatment with BMSCs elevated survival rates in a dose-dependent manner as compared to the LPS group (Fig. 5).

### BMSCs inhibit LPS-stimulated PBMCs proliferation

**Fig. 6:**
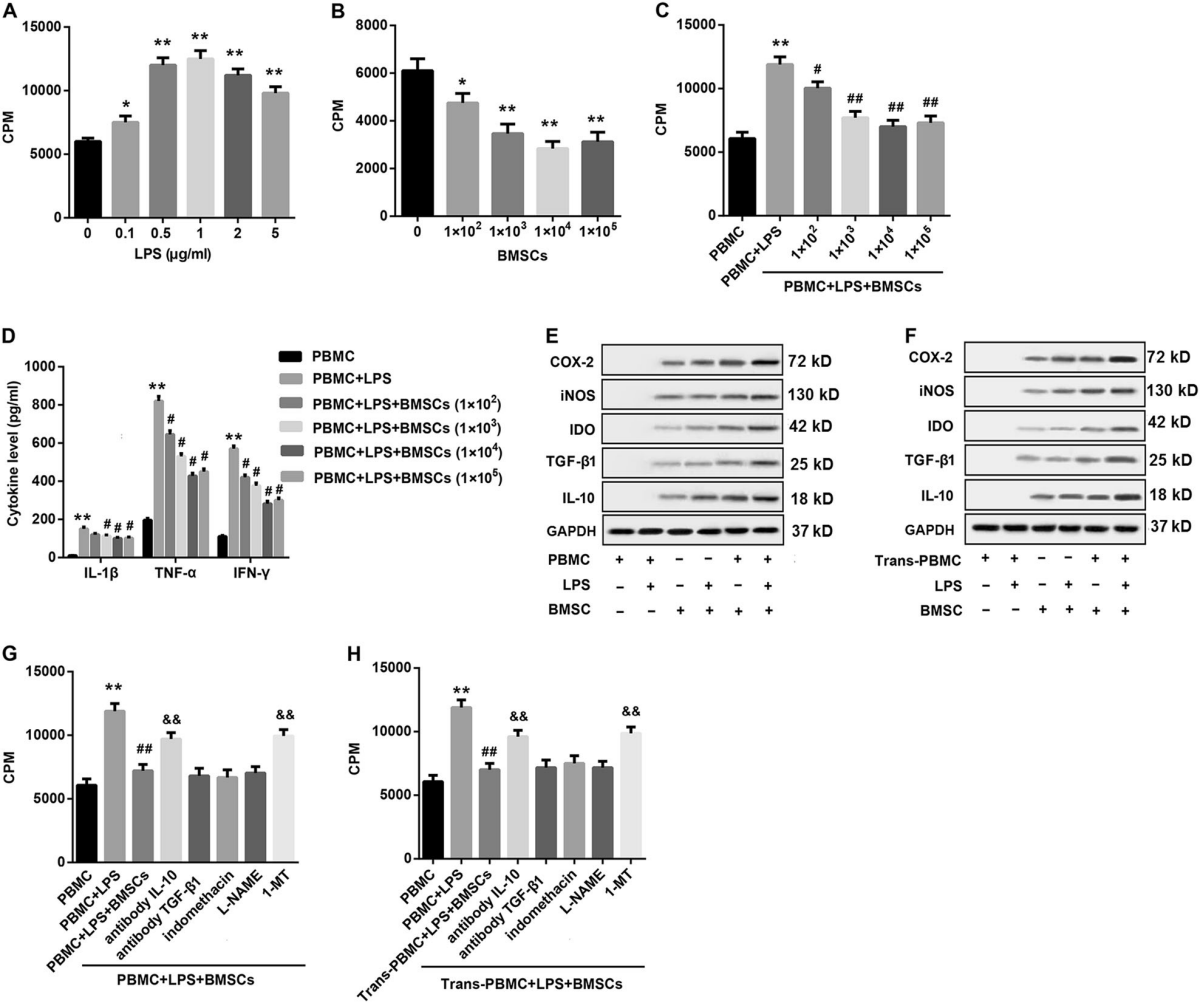
Soluble mediators were involved in BMSCs-mediated inhibition of LPS-stimulated PBMCs. **a** Treatment with LPS (final concentrations, 0, 0.1, 0.5, 1, 2, and 5 μg/ml) significantly stimulated proliferation capacity of PBMCs (100 μl, 2 × 10^6^/ml) in vitro. **b** Treatment with allogeneic BMSCs of various doses (0, 1 × 10^2^, 1 × 10^3^, 1 × 10^4^, and 1 × 10^5^) significantly suppressed proliferation capacity of PBMCs (100 μl, 2 × 10^6^/ml) in vitro. **c** BMSCs with various doses (0, 1 × 10^2^, 1 × 10^3^, 1 × 10^4^, and 1 × 10^5^) significantly inhibited the LPS (0.5 μg/ml)-stimulated proliferation capacity of PBMCs (100 μl, 2 × 10^6^/ml). **d** BMSCs with various doses (0, 1 × 10^2^, 1 × 10^3^, 1 × 10^4^, and 1 × 10^5^) significantly reduced LPS (0.5 μg/ml)-stimulated release of pro-inflammatory cytokines (TNF-α, IFN-γ, IL-1β) from PBMCs. **e** Western blot was performed to evaluate protein levels of COX-2, iNOS, IDO, TGF-β1, and IL-10 in BMSCs (1 × 10^4^) co-cultured with PBMCs (2 × 10^5^) in cell–cell contact condition under LPS stimulation or not. **f** Western blot was performed to detect protein levels of COX-2, iNOS, IDO, TGF-β1, and IL-10 in BMSCs (1 × 10^4^, lower) under transwell co-culture with PBMCs (2 × 10^5^, upper) under LPS stimulation or not. β-actin served as the loading control. **g** ^3^H-TdR incorporation assay was carried out to examine the proliferation capacity of PBMCs pre-treated with the indicated conditions under direct contact co-culture of PBMCs (2 × 10^5^) and BMSCs (1 × 10^4^) in each group. **h** ^3^H-TdR incorporation assay was carried out to examine the proliferation capacity of PBMCs pre-treated with the indicated conditions under Transwell co-culture of PBMCs (2 × 10^5^, upper) and BMSCs (1 × 10^4^, lower) in each group. β-actin served as the loading control. Data were expressed as mean ± standard deviation from three independent experiments. ^*^P < 0.05, ^**^P < 0.01, *vs*. PBMCs; ^#^*P* < 0.05, ^##^*P* < 0.01, vs. PBMCs + LPS; ^&&^*P* < 0.01, vs. PBMCs + LPS + BMSCs or Trans-PBMCs + LPS + BMSCs

The proliferative capacity of PBMCs from rats which underwent co-culture with various doses of LPS alone or BMSCs alone was measured by ^3^H-TdR incorporation assay. The uptake of the thymidine analog BrdU on PBMCs revealed that co-culture with various concentrations of LPS for 72 h significantly stimulated the proliferation of PBMCs, and the proliferation of 1 μg/ml LPS group reached the highest (Fig. 6a). In contrast with LPS, treatment with allogeneic BMSCs with various doses for 72 h significantly suppressed the proliferation capacity of PBMCs (Fig. 6b). Next, we explored whether BMSCs had immunosuppressive effects on the proliferation of T lymphocytes in response to LPS stimulation in vitro. To this end, the isolated PBMCs pre-treated with various doses of BMSCs were co-cultured with LPS (0.5 μg/ml) for 72 h in vitro. Data demonstrated that pre-treatment with BMSCs significantly inhibited the LPS-stimulated proliferation of PBMCs, and 1 × 10^4^ BMSCs reached the strongest suppression effect (Fig. 6c).

### BMSCs reduce the LPS-stimulated release of inflammatory cytokine from PBMCs

Next, the changes of inflammatory cytokines released by lymphocyte in medium also measured. We found that LPS significantly stimulated the release of inflammatory factors from PBMCs. However, if PBMCs were co-cultured with BMSCs, the LPS-stimulated release of inflammatory cytokines from PBMCs was significantly inhibited. In addition, levels of these cytokines in 1 × 10^4^ BMSCs group were the lowest (Fig. 6d).

### Soluble mediators are involved in BMSCs-mediated inhibition of LPS-stimulated PBMCs

As shown in Fig. 6e, a significant increase in the protein expression levels of the soluble mediators, including COX-2, iNOS, IDO, TGF-β1, and IL-10, in BMSCs co-cultured with PBMCs in cell–cell contact condition under LPS stimulation was observed compared with that in BMSCs alone. To determine whether cell–cell contact between BMSCs and PBMCs was required for the suppression, PBMCs were cultured in the upper chamber of a Transwell separated by a semipermeable membrane from BMSCs cultured in the lower chamber. A similar result was also found in Transwell culture condition where cell–cell contact between BMSCs and PBMCs was prevented, indicating that soluble factors were operative (Fig. 6f).

We next determined the role of soluble mediators in the BMSCs-mediated suppression of PBMCs proliferation. To this end, BMSCs were pre-treated with neutralizing antibodies for IL-10 and TGF-β1 that are known to be produced by BMSCs, or chemical antagonists for COX-2 (indomethacin), iNOS (L-NAME), or IDO (1-MT) for at least 2 h, followed by co-culture with PBMCs in cell–cell contact and Transwell culture conditions under LPS stimulation for 72 h, and then PBMCs numbers were counted. Data demonstrated that BMSCs-suppressed LPS-stimulated proliferation of PBMCs both in cell–cell contact and Transwell culture conditions, indicating that the BMSCs-mediated suppression of PBMCs proliferation did not require direct cell-to-cell contact. The finding suggested the possibility that it was mediated through some soluble factors. Meanwhile, pre-treatment with neutralizing antibody for IL-10 and the specific IDO inhibitor 1-MT significantly reversed the BMSCs-mediated suppression of PBMCs proliferation in the presence of LPS in both cell–cell contact and Transwell culture conditions. However, treatment with neutralizing antibody for TGF-β1, antagonists for COX-2 and iNOS, failed to reverse the BMSCs-mediated suppression on PBMC proliferation. These results indicated that IL-10 and IDO, rather than TGF-β1, COX-2, or iNOS, may contribute, at least in part, to BMSCs-mediated inhibition of PBMCs proliferation (Fig. 6g, h).

### IFN-γ-induced upregulation of IDO and IL-10 contributes to BMSCs-mediated inhibition of LPS-stimulated PBMCs proliferation

**Fig. 7:**
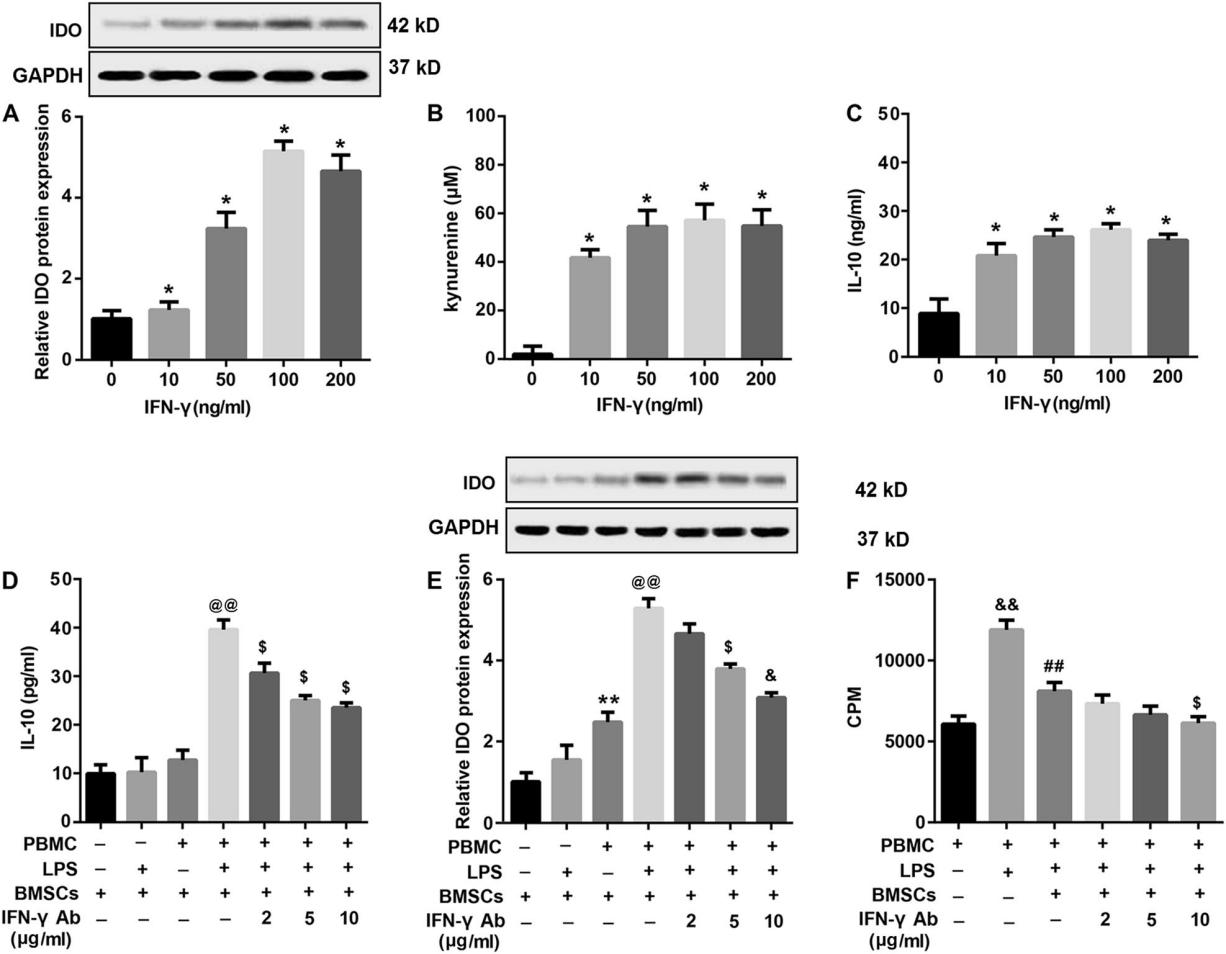
Upregulation of IFN-γ-induced IDO and IL-10 contributed to BMSCs-mediated inhibition of LPS-stimulated PBMCs proliferation. BMSCs were stimulated with increasing concentrations of IFN-γ (0, 10, 50, 100, and 200 ng/ml) for 24 h. **a** Then the protein expression of IDO in BMSCs was determined by western blot and statistical analysis of relative protein expression was shown. **b** IDO activity was analyzed by measuring the concentration of kynurenine in the culture medium using an HPLC technique. **c** IFN-γ-treated IL-10 secretion in the supernatants was determined using ELISA. Next, PBMCs (2 × 10^5^) were pre-treated with different concentrations of specific IFN-γ neutralizing antibody (2, 5, and 10 μg/ml) for 2 h, followed by co-culture with BMSCs (1 × 10^4^) under cell–cell contact conditions in the presence of LPS (0.5 μg/ml). Then **d** the concentrations of IL-10 in the supernatants and **e** IDO protein expression in PBMCs were examined. **f** The proliferation capacity of PBMCs was detected by ^3^H-TdR incorporation assay. β-actin served as the loading control. Data were expressed as mean ± standard deviation from three independent experiments. ^*^P < 0.05, ^**^P < 0.01, vs. 0 ng/ml IFN-γ or BMSCs; ^@@^*P* < 0.01, vs. PBMCs + BMSCs; ^$^*P* < 0.05, vs. PBMCs + LPS + BMSCs; ^&&^*P* < 0.01, vs. PBMCs; ^##^*P* < 0.01, vs. PBMCs + LPS

The inflammatory cytokine IFN-γ has been shown to be capable of regulating the immunomodulatory functions of BMSCs via upregulation of some immunosuppressive factors, including IL-10 and IDO^18^. Data revealed that IFN-γ treatment significantly upregulated protein expression levels of IDO protein expression in BMSCs in a dose-dependent manner (Fig. 7a). In addition, functional assays revealed that the concentration of kynurenine, a metabolic product of IDO, dose-dependently increased in supernatants of BMSCs in response to IFN-γ stimulation (Fig. 7b), indicating that the BMSCs-induced IDO molecule was active. Furthermore, IFN-γ also stimulated IL-10 secretion by BMSCs in a dose-dependent manner (Fig. 7c).

Accordingly, we speculated that the increased IL-10 secretion and IDO expression by BMSCs may be attributed to an increased IFN-γ production by LPS-stimulated PBMCs. To this purpose, we pre-treated PBMCs with increasing concentrations of IFN-γ-neutralizing antibody followed by co-culture with BMSCs in the presence or absence of LPS for 24 h. We found that that treatment with IFN-γ-neutralizing antibody significantly inhibited IL-10 secretion (Fig. 7d) and IDO expression (Fig. 7e) by BMSCs upon co-culture with LPS-stimulated PBMCs. In addition, our results showed that IFN-γ neutralizing antibody led to further inhibition of BMSCs-suppressed proliferation of PBMCs under LPS stimulation (Fig. 7f). These results indicated that IFN-γ contributed to BMSC-mediated inhibition of PBMCs through the upregulation of IL-10 and IDO expression.

## Discussion

Pro-inflammatory cytokines such as TNF-α, IFN-γ, and IL-1β play critical roles in the pathologic processes, leading to the microthrombus formation in DIC models^32,33^. Accordingly, we speculated that reduced secretion of these pro-inflammatory factors would be beneficial for the prevention and treatment of DIC. As described previously, BMSCs could regulate these important pro-inflammatory cytokines via a variety of effector mechanisms on immune cells. However, whether BMSCs are helpful in LPS-induced DIC rat models remains unknown. Hence, we investigated the effectiveness of BMSCs injection in ameliorating LPS-induced DIC.

Intravenous infusion of LPS is frequently used to induce DIC in experimental animal models, which importantly mimic clinical DIC in patients with sepsis^34^. In this study, we generated a rat model of DIC by LPS injection via the tail vein to investigate the protective effect of BMSCs on coagulation system and organ function. In this study, the LPS infusion group displayed a significantly lower plasma PLT quantities and Fib levels, significantly higher d-dimer levels, prolonged times of APTT and PT. Furthermore, HE staining and PTAH staining indicated the formation of microvascular thrombosis and cell injury in the LPS group. Together, these results indicated that the LPS-induced DIC rat model was established successfully according to the criteria for the diagnosis of DIC.

More importantly, we found that BMSCs markedly alleviated systemic intravascular coagulation, as evidenced by increased PLT counts and Fib concentrations, decreased plasma d-dimer levels, and shortened APTT and PT as compared with the LPS group. Furthermore, BMSCs pre-treatment exerted protective effects on organ function, as revealed by lower plasma levels of organ function-associated indicators (ALT, Cr, and CK-MB), and reduced formation of microvascular thrombosis and alleviated organ injury (in heart, liver, and kidney). Moreover, the reduced 24-hour survival rates in the LPS group were increased by BMSCs pre-treatment. In addition, the BMSCs pre-treatment groups displayed reduced plasma levels of ET that served as a potent vasoconstrictor and an aggravating factor of DIC^35,36^, which indicated that BMSCs pre-treatment alleviated the damage of endothelial cells and combat DIC progression. Collectively, these observations supported the notion that BMSCs pre-treatment ameliorated tissue destruction in LPS-induced DIC rat model. Our results also revealed that more protective effects occurred with more BMSCs pre-treatment, and the best protective effects were achieved when the number of BMSCs was between 1 × 10^5^ and 1 × 10^6^.

In addition, our studies showed that BMSCs pre-treatment significantly reduced the plasma levels of pro-inflammatory cytokines TNF-α, IFN-γ, and IL-1β. The regulation of cytokines might represent a vital mechanism underlying BMSCs-mediated protection. Furthermore, cytokines and inflammatory mediators released from lymphocytes play important roles in the progression of DIC. Thus, we speculated that BMSCs might combat DIC by regulation of immunocytes in DIC models. Moreover, the proliferation and activation of lymphocytes play an important role in the immune response^37^. Hence, investigation of the altered proliferation of PBMCs is of great significance. In the last few years, it has become clear that BMSCs also possess immunomodulatory properties. The mechanisms involved in the immunomodulatory activity of BMSCs on T lymphocytes are still partially obscure.

BMSCs play a key role in regulating the maturation and proliferation as well as functional activation of lymphocytes. Nicola et al.^11^ proposed that allogeneic BMSCs strongly suppress T-lymphocyte proliferation, which was consistent with our observations. As far as the mechanism underlying BMSC-mediated suppression of T-lymphocyte proliferation is concerned, data reported so far remains controversial. Is it dependent on direct contact or secretion of cytokines? Which cytokine? How? The mainstream opinion is that the suppressive activity of BMSCs on PBMCs proliferation does not depend on cell-to-cell contact^7,11,18,38^, which is in agreement with our findings. As for involved cytokines, a growing body of evidence has indicated that soluble factors such as TGF-β1, HGF, IL-10, IDO, nitric oxide, human leukocyte antigen-G5 (HLA-G5), and prostaglandin E2 (PGE2), play critical roles in MSC-mediated immunosuppression^11,16–18,39^. However, the relative contribution of these soluble factors to the immunosuppressive effects of MSCs varies and remains controversial under different experimental conditions. For instance, a previous study indicated that the inhibition of T-lymphocyte proliferation was not due to induction of apoptosis and was likely due to the production of soluble factors TGF-β1 and HGF^11^. But in other studies^40^, TGF-β1 and IL-10 by BMSCs appeared not to account for the BMSCs-mediated suppression of PBMCs proliferation. Moreover, controversies about the role of HGF and PGE2 in MSC-mediated immunosuppression have also been reported^18,39,41^. In our study, we found that the suppressive activity of BMSCs on PBMCs proliferation was abrogated by blocking IL-10 and IDO but not TGF-β1, iNOS, or COX-2, both under both cell–cell contact and Transwell conditions with LPS stimulation. These results indicate that IL-10 and IDO might contribute to BMSCs-mediated immunosuppression of PBMCs proliferation.

Increasing studies have shown that IFN-γ serves as critical feedback signal molecules in the cross-talk between immune cells and MSCs cells^18^. Upon activation, immune cells secrete a high amount of inflammatory cytokines, especially IFN-γ, which may subsequently stimulate MSCs to express various immunosuppressive molecules, such as IDO, resulting in a negative feedback inhibition of inflammatory cell responses in terms of proliferation and cytokine secretion^7,17^. In addition, the crucial role of IFN-γ in BMSCs-driven suppression was further supported by several studies. For example, Krampera et al.^7^ indicated that the suppressive activity of BMSCs on T lymphocytes proliferation required the presence of IFN-γ produced by activated T cells and NK cells. And even activated B cells that were unable to produce IFN-γ became susceptible to the suppressive activity of BMSCs in the presence of exogenously added IFN-γ. In our study, we demonstrated that IFN-γ stimulation significantly increased the level of functional IDO and enhanced IL-10 secretion by BMSCs. Furthermore, in response to LPS stimulation, BMSCs exhibited enhanced secretion of IFN-γ and IL-10 as well as IDO expression. Moreover, co-culture with BMSCs resulted in suppression of LPS-stimulated proliferation of PBMCs and secretion of IFN-γ and IL-10 as well as IDO expression. More importantly, blocking IFN-γ led to a significant and dose-dependent reduction of IL-10 secretion and IDO expression, as well as an abrogation of BMSCs-mediated inhibition of PBMCs proliferation. Taken together, these findings indicate that IFN-γ promotes the immunomodulatory activity of BMSCs, which in turn suppress PBMCs proliferation. The ability of IFN-γ to induce the immunosuppressive effect of BMSCs on PBMCs proliferation appeared to be, at least in part, related to the enhancement of the IDO activity and secretion of IL-10.

Many studies investigated the therapeutic effects of BMSCs in injury-models induced by LPS. For example, Yagi et al.^42,43^ demonstrated that intramuscular transplantation of BMSCs immediately after the LPS induction decreased inflammatory cytokines and attenuated multiple organ injury in LPS-induced endotoxemic rats. Zhang et al.^44^ showed that BMSCs transplantation attenuated d-galactosamine/LPS-induced acute liver failure in a rat model. Zhao et al.^45^ have also suggested that intravenous transplantation of BMSCs into the injured rats shortly following LPS challenge alleviated local inflammation and severity of acute lung injury. Our findings from this current study revealed that BMSCs pre-treatment ameliorated inflammation-related tissue destruction in LPS-induced DIC rat model. However, the protective effects of transplanted BMSCs were unclear. It is worth noting that a previous study has confirmed that administering BMSCs to mice either before or shortly after inducing sepsis reduced mortality and improved organ function^22^. This study provided supporting evidence for the protective effects of transplanted BMSCs in DIC, which requires our further investigation.

## Conclusion

In conclusion, the data presented in this paper demonstrated that BMSCs pre-treatment effectively inhibited intravascular coagulation, ameliorated inflammation-related tissue destruction, and improved the 24-hour survival rates of LPS-induced DIC model rats. The protection of BMSCs could be due to their capabilities to suppress production of inflammatory mediators and  to regulate immune tolerance, which render BMSCs a promising source for stem cell-based therapeutic approaches in inflammation-related DIC.

## Electronic supplementary material

Supplementary Figure 1

Supplementary figure legends
