# Supplementary figures and images for: BMSCs pre-treatment ameliorates inflammation-related tissue destruction in LPS-induced rat DIC model
Source: Cell Death Dis. 2018 Oct 3;9(10):1024. doi: 10.1038/s41419-018-1060-5 (PMC6170466; doi:10.1038/s41419-018-1060-5)

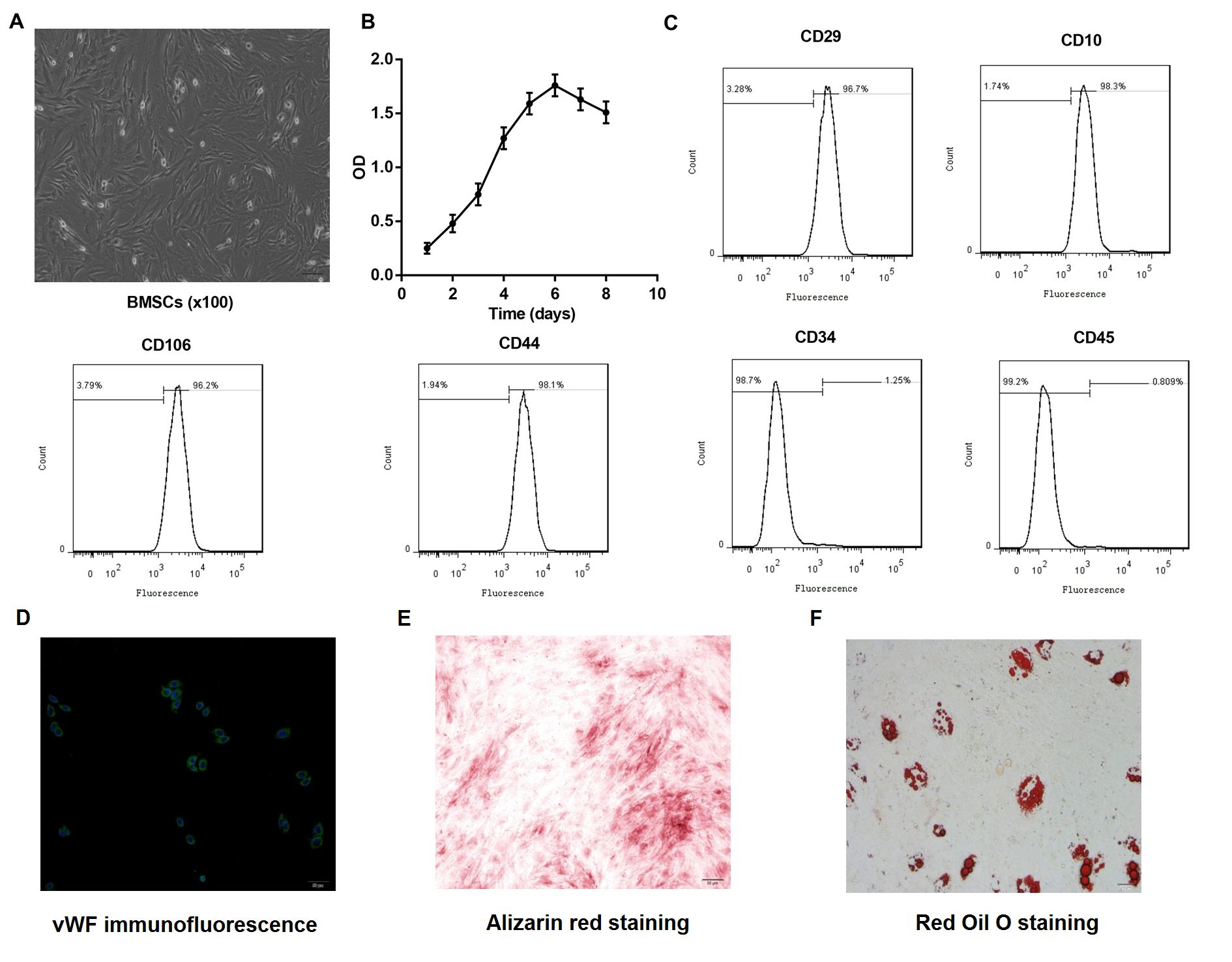

Supplement: Supplementary file 1 — Supplementary Figure 1 [file 41419_2018_1060_MOESM1_ESM.tif]
