# Supplementary material for: BMSCs pre-treatment ameliorates inflammation-related tissue destruction in LPS-induced rat DIC model
Source: Cell Death Dis. 2018 Oct 3;9(10):1024. doi: 10.1038/s41419-018-1060-5 (PMC6170466; doi:10.1038/s41419-018-1060-5)
Supplement: Supplementary file 2 — Supplementary figure legends [file 41419_2018_1060_MOESM2_ESM.docx]

**Supplementary Figure 1. Culture and characterization of BMSCs.** BMSCs were isolated from Wistar rats and expanded *in vitro*. **(A)** Phase-contrast microscopy of the spindle-shaped morphology of rat BMSCs in the fourth passage (magnification, ×100). **(B)** The growth curve of the cells cultured for 1-8 days evaluated by MTT assay was drawn. **(C)** Analysis of the immunophenotype of BMSCs by flow cytometry. BMSCs were positive for CD29, CD10, CD106, and CD44, while negative for CD34, CD45. **(D)** Endothelial differentiation of BMSCs revealed forming of the phenotype of von Willebrand factor (vWF) by immunofluorescence (magnification, ×100). **(E)** Osteogenic differentiation of BMSCs revealed forming of calcium deposits by Alizarin Red staining (positive: red, magnification, ×400). **(F)** Adipogenic differentiation of BMSCs revealed forming of lipid vacuoles by Oil Red O staining (positive: red, magnification, ×100). Results are representative graphs of three independent experiments.
